# The ineffective emotion regulation of deaf college students: an ERP study

**DOI:** 10.3389/fnhum.2024.1445397

**Published:** 2024-09-11

**Authors:** Qi Dong, Le Sun, Xue Du

**Affiliations:** Key Laboratory of Applied Psychology, Chongqing Normal University, Chongqing, China

**Keywords:** deaf college students, emotion regulation, cognitive reappraisal, expression suppression, ERP, LPP

## Abstract

**Introduction:**

Deaf students have more difficulties with emotion regulation due to their hearing loss. They are suffering higher socio-emotional risk than the hearing person. But there are few studies explored the neural mechanisms of impaired emotion regulation in the deaf college students.

**Methods:**

Thirty hearing college students and 27 deaf college students completed the emotion regulation task while recording ERP data and subjective emotion intensity.

**Results:**

Behavioral results found that deaf college students had higher emotional experience intensity compared to healthy controls. The ERP results showed the deaf college students had lower LPP amplitudes both using reappraisal and suppression strategies. Moreover, the LPP of expression suppression was associated with the increase of depression scores among deaf college students.

**Discussion:**

Deaf college students may have impaired emotion regulation so that they are more accustomed to using expression suppression strategies to regulate their negative emotions which lead to high risk to be depression.

## 1 Introduction

The World Health Organization (WHO) estimates that approximately 360 million people in the world currently have disabling hearing impairments (Lam et al., 2016). Deaf people are not exposed to health-related information to the same extent as hearing adults (Barnett et al., 2011), they may have lower health literacy (Kushalnagar et al., 2018), which puts them at a disadvantage in preventing negative health outcomes and engaging in healthy lifestyles. For example, they are suffering higher socio-emotional risk than the hearing person (Adibsereshki et al., 2019). We have known that the regulation of responses to emotional stimuli is critical to mental health (Gross and Muñoz, 1995; Paul et al., 2016).

Emotion regulation is a process that enables us to adjust the intensity, the timing, and the way we experience and express emotions (Gross, 1998). As we know, emotion regulation comprises situation selection/modification, attentional deployment, cognitive change, at each emotion regulation point, there are certain strategies to voluntarily modulate emotions (Gross, 1998; Palmer and Alfano, 2017). Previous studies focused on the two most common and valuable strategies for emotion reduction: cognitive reappraisal and expression suppression (Ding et al., 2021; Gross, 2002; Kobylinska and Kusev, 2019). As antecedent-focused emotion regulation, cognitive reappraisal refers to the early modification of emotions before emotional responses are activated (Horan et al., 2013; Troy et al., 2018), which generate positive outcomes, such as enhancing working memory capacity (McRae et al., 2012), reducing negative emotions (Memedovic et al., 2010). On the other hand, as response-focused emotion regulation, expression suppression refers to the suppression of emotional expression behaviors that will occur or are occurring (Freitas et al., 2022). Resulting in negative consequences, for example, debilitating effect on intensity of negative emotions (Hofmann et al., 2009), and learning performance (Ben-Eliyahu and Linnenbrink-Garcia, 2015).

In addition to behavioral measurements in emotion regulation tasks, the late positive potential (LPP) component of the event-related potential (ERP) may serve as a useful index of emotion regulation at the neural level (Hajcak et al., 2010). The LPP is an established sensitive and reliable measure of processing of emotionally charged stimuli (Olofsson and Polich, 2007), beginning approximately 300 ms after the initiation of the stimulus, reaching a maximum amplitude 400–700 ms after the stimulus, lasting for hundreds of milliseconds, with the maximum intensity usually in the central parietal region (Paul et al., 2013).

The magnitude of LPP is manipulated by various emotional regulations and its duration can quantify the emotional response and regulation (Hajcak et al., 2010). Numerous studies have shown that the LPP amplitude induced by emotional stimuli decreased after using cognitive reappraisal and expression suppression (Desatnik et al., 2017; Moran et al., 2013; Moser et al., 2006). Moreover, LPP has been found to be a suitable indicator of emotion dysregulation. For example, abnormal LPP responses have been observed in individuals with intrinsic psychopathology (e.g., depression, schizophrenia) and drug use disorders (Beauchaine et al., 2020; Ding et al., 2021; Hudak et al., 2022; Martin et al., 2017).

Previous behavioral results showed the deaf college students have significantly higher difficulties in overall emotional regulation than hearing college students (Zhang et al., 2012). To address the gap in the research on the neural mechanisms of impaired emotion regulation in deaf college students, the present study aimed to explore whether there are behavioral and neurological obstacles in the use of emotion regulation strategies in deaf college students. Based on previous findings, the research not only focused on behavioral tasks, but also applied EEG techniques to explore whether there are differences between deaf college students and hearing college students in terms of their emotion regulation ability.

## 2 Methods and materials

### 2.1 Participants

For the sample size, we used G*Power 3.1 for sample size estimation (Faul et al., 2009), the minimum sample size required for this study was 36 to achieve a test power of 0.95 (α = 0.05) at a medium effect size (0.25). To prevent the sample size from being insufficient due to missing data, we expanded the sample size by 20%, resulting in a sample size of at least 44 persons. Fifty seven students (32 males and 25 females) from Chongqing Normal University were paid to participate in the experiment, including 30 hearing college students as the healthy control group (age: 20.30 ± 1.97 years, 15 males and 15 females) and 27 deaf college students as the deaf student group (age: 20.44 ± 1.60 years, 17 males and 10 females). All the deaf participants were college students enrolled in the Department of Special Education of the Normal University. They entered the university through a single examination and a single enrollment for undergraduate education, had access to the same educational resources as hearing college students and were also eligible for master’s degree programs. The deaf college students were sign language users, with an average hearing loss of 71 decibels or more in both ears. All participants were right-handed, had normal bare or corrected vision, no color blindness, and had not participated in similar experimental studies. Each participant signed the appropriate informed consent prior to the experiment. After all experiments were completed, participants were asked to fill out the Beck Depression Inventory-II (BDI-II) (Yang et al., 2014) and the Emotion Regulation Questionnaire (ERQ) (Wang et al., 2007). Table 1 provides detailed participant information. The study has obtained the informed consent of all participants, and they are informed of the ethical principles of voluntary participation. The study complies with the Declaration of Helsinki and received ethical clearance from the ethical committee of Institute of Psychology, Chongqing Normal University.

**Table 1:** Demographic and clinical characteristics of subjects (*N* = 57).

Measure	Healthy control group	Deaf student group	Between-groups *p* value
Subjects	30	27	
Sex (male/female)	15/15	17/10	0.325
Age	20.30 (1.97)	20.44 (1.60)	0.764
**ERQ**			
Cognitive reappraisal	31.33 (5.74)	30.89 (5.00)	0.758
Expression suppression	7.57 (2.49)	10.74 (3.02)	<0.001

### 2.2 Questionnaires

#### 2.2.1 Beck depression inventory-II

The Beck Depression Inventory-II (BDI-II), developed by Beck et al. (1996), and revised by Wang et al. (2011). The scale was used to assess the severity of depressive symptoms in the past 2 weeks, and consisted of 21 items, each of which was rated on a scale of 0 to 3. The total score of the scale was the sum of 21 items, and the total score range was 0 to 63 points. According to the demarcated scores of the original scale, the total score of 0 ~ 13 was classified as no depression, 14 ~ 19 as mild depression, 20 ~ 28 as moderate depression, and 29 ~ 63 as severe depression. This scale has good reliability and validity in China (Jiang and Yang, 2020; Wang et al., 2011), and can be used as a self-assessment tool to assess the severity of depressive symptoms.

#### 2.2.2 Emotion regulation questionnaire

The Emotion Regulation Questionnaire was developed by Gross and John (2012), revised by Wang et al. (2007). The questionnaire is a 7-point Likert scale, requiring subjects to give answers ranging from completely disagree (1) to completely agree (7) for each item according to their own circumstances. The questionnaire included 10 items, with items 1, 3, 5, 7, 8, and 10 measuring reevaluation, and items 2, 4, 6, and 9 measuring expression suppression. Each dimension includes at least one item that measures regulation of positive emotions and one item that measures regulation of negative emotions. The expression inhibition sample was entitled “I do not show my emotions” and the reappraisal sample was entitled “I change my perception of the situation when I want to feel a more positive emotion.” This scale has good reliability and validity and cross-cultural stability (Wang et al., 2007).

### 2.3 Stimuli

#### 2.3.1 Faces

Forty sad images were chosen from the Chinese Facial Affective Picture System (CFAPS) (Bai et al., 2005), with equal numbers of male and female pictures. According to the data from the CFAPS database, there was no significantly difference in the arousal of the chosen images. Participants were seated in a quiet room with their eyes 70 cm from the screen and horizontal and vertical view angles below 5°. All images were presented at the same brightness and contrast on a black background (central presentation).

### 2.4 Experimental design and procedure

**Figure 1:**
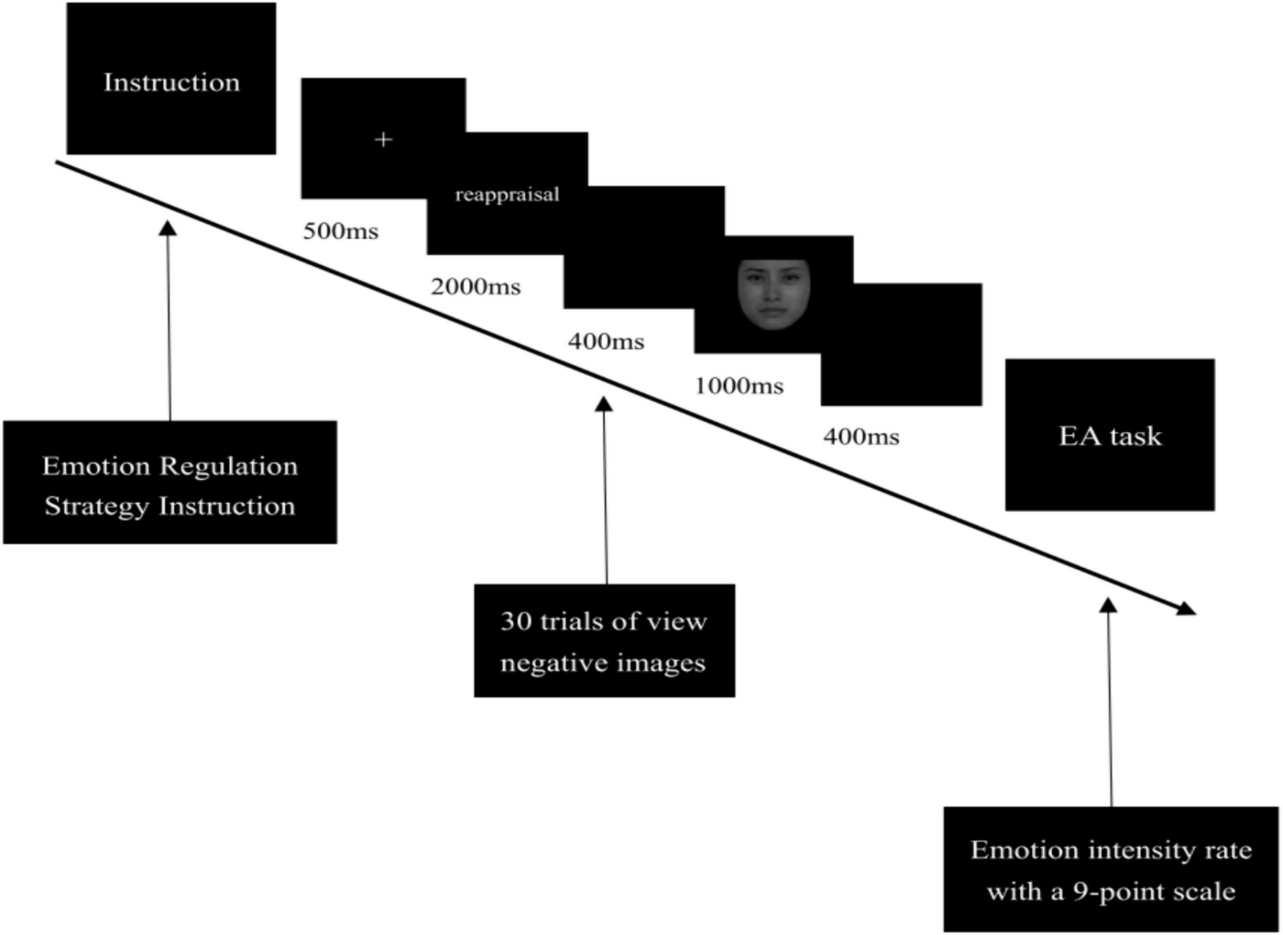
The sequence of the task in one single block.

The program was programmed and demonstrated by E-prime 3.0. The task was designed with a classical emotion regulation task (Desatnik et al., 2017; Moser et al., 2006). The experimental task was divided into three conditions, passive view, reappraisal condition, and suppression condition, for a total of six blocks. Each condition consists of two successive blocks, and each block ended with one trial of the emotion assessment task (EA task). As shown in Figure 1, in the passive view condition, participants were asked to view the images directly, and the cognitive reappraisal negative and expression suppression negative conditions first presented the corresponding instructions to prompt the participants what to do next. Cognitive reappraisal strategies require participants to adopt a transcendent, unemotional and neutral attitude and to reappraise the images in a way that is unrelated to their emotions. Expression suppression strategy requires participants to suppress their emotions and view the images calmly without showing their emotions. After the instruction is presented, a 500 ms fixation cross appeared in the center of the screen, followed by a 2,000 ms presentation of cue words (cognitive reappraisal cue word: reappraisal, expression suppression cue word: suppression), then a 400 ms blank screen, next a 1,000 ms image was presented, and an empty 400 ms screen. After all the images were presented, the participants were asked to rate the intensity of the emotion they were experiencing in the moment on a scale of 1–9. A “1” means “very calm” and a “9” means “very intense.”

The pseudo-randomized design was used in this study, and passive view was placed before reappraisal negative and suppression negative in order to avoid the effect of regulation condition on neutral condition. The order between reappraisal negative and suppression negative was balanced.

### 2.5 EEG recording

EEG was continuously recorded using a 64-channel Ag-AgCl electrode cap (Brain Products) based on the 10–20 International System. Bilateral mastoids were used as reference electrodes (bilateral mastoids were averaged for reference) and electrodes were placed laterally in both eyes to record horizontal electro-oculography and above and below the left eye to record vertical electro-oculography. Scalp impedance for each electrode was kept below 5KΩ. EEG data preprocessing was conducted using MATLAB (version 9.3.0.713579 [R2017b]; The MathWorks, Inc.) script set developed by the authors, containing both original and EEGLAB functions (Delorme and Makeig, 2004), while ERP analysis was performed in the Psychophysiology Toolbox (Bernat et al., 2005). EEG signal was filtered using a range of 0.05–100 Hz and sampled using the rate of 500 Hz. The data is processed offline after completion of continuous recording of the EEG. We used ERPLAB to filter the data, set the band-pass as 0.1–50 Hz, and blink and eye movement artifacts were eliminated after independent component analysis. The offline analysis period was 1,200 ms, including 200 ms before the presentation of the feedback stimulus (as a baseline) and 1,000 ms after the presentation for analysis. Segments were manually inspected to ensure exclusion of trials with artifact.

### 2.6 Data analysis

We analyzed emotional experience intensity rating according to three different conditions in the experimental procedure. According to the literature (Dennis and Hajcak, 2009; Hajcak et al., 2010; Li et al., 2022), the LPP was defined as the mean amplitude in three times windows following the stimulus onset: early (350–600 ms), middle (600–1,000 ms), and late (1,000–1,500 ms) time windows. And visual inspection of the topographical distribution of grand averaged ERP activity, LPP was measured as the average activity of CPz, CP1, CP2 and the average amplitudes calculation window was 420–620 ms. We measured averaged amplitudes, instead of peak amplitudes.

The Greenhouse–Geisser correction was used to compensate for sphericity violations. Least significant difference (LSD) was applied for *post hoc* testing of main effects. Partial eta-squared (*η_p_*^2^) was reported as indicator of the effect size in ANOV A tests. All these statistical analyses were conducted with SPSS 23.0 software.

## 3 Results

### 3.1 Behavioral data

The difference in ERQ scores between the two groups of subjects was statistically significant. As expected, the deaf college student group had significantly higher expression suppression scores than the control group (*t*_(55)_ = 4.348, *p* < 0.001) (Table 1).

**Figure 2:**
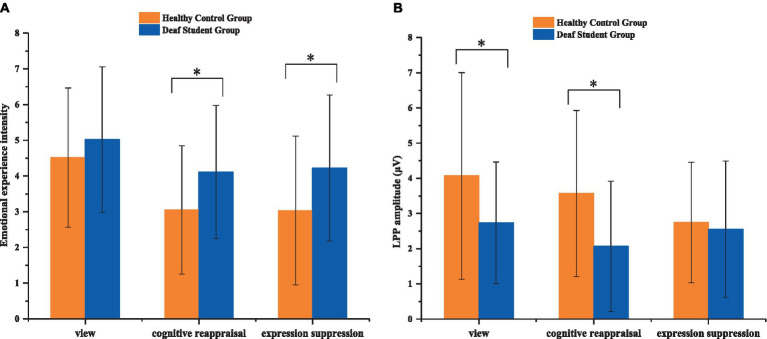
Emotional experience intensity **(A)** and LPP amplitude **(B)** during the task (*p < 0.005). Error bars represent standard errors.

Emotional experience intensity rating scores were analyzed using a 2 (groups: deaf student group/healthy control group) × 3 (condition: passive view/reappraisal negative/suppression negative) repeated-measure ANOVA which revealed a significant main effect of group (*F*_(1, 55)_ = 4.350, *p* = 0.042, *η_p_*^2^ = 0.073), whereby the deaf student group elicited larger emotional experience intensity (*M* = 4.451, SD = 0.319) than the control group (*M* = 3.533, SD = 0.303) (Table 2). Results also revealed a significant main effect of condition (*F*_(2, 110)_ = 6.987, *p* = 0.002, *η_p_*^2^ = 0.206). Specifically, reappraisal negative (*M* = 3.581, SD = 0.242) and suppression negative (*M* = 3.628, SD = 0.274) were associated with a lower emotional experience intensity score during the emotion regulation task than scores associated with passive view (*M* = 4.768, SD = 0.264), and two emotion regulations showed no difference (*p* > 0.05). The interaction between factors was not significant. To examine the differences between groups, analysis using independent samples t-tests found that for reappraisal negative (*t*_(55)_ = 2.188, *p* = 0.033) and suppression negative (*t*_(55)_ = 2.170, *p* = 0.034) scores, the deaf college group was significantly higher than the control group (Figure 2A).

**Table 2:** Emotional experience intensity and LPP amplitudes during the task.

Group	Passive view	Reappraisal negative	Suppression negative
**Emotional experience intensity, mean (SE)**			
Healthy control group	4.52 (1.95)	3.05 (1.80)	3.03 (2.08)
Deaf student group	5.02 (2.04)	4.11 (1.86)	4.22 (2.04)
**LPP amplitudes, mean (SE)**			
Healthy control group	4.07 (2.94)	3.57 (2.36)	2.74 (1.71)
Deaf student group	2.73 (1.73)	2.07 (1.85)	2.55 (1.93)

### 3.2 ERP results

#### 3.2.1 LPP

**Figure 3:**
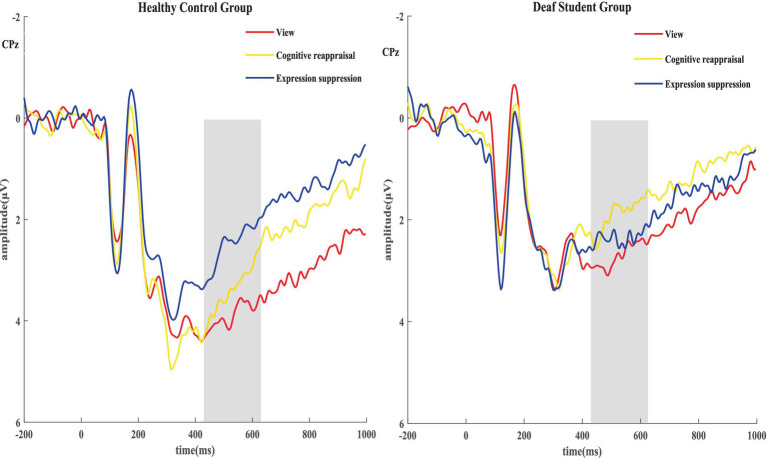
Grand average event-related potential at the CPz site during three conditions for the deaf student group and the healthy control group (shading indicates LPP time windows).

**Figure 4:**
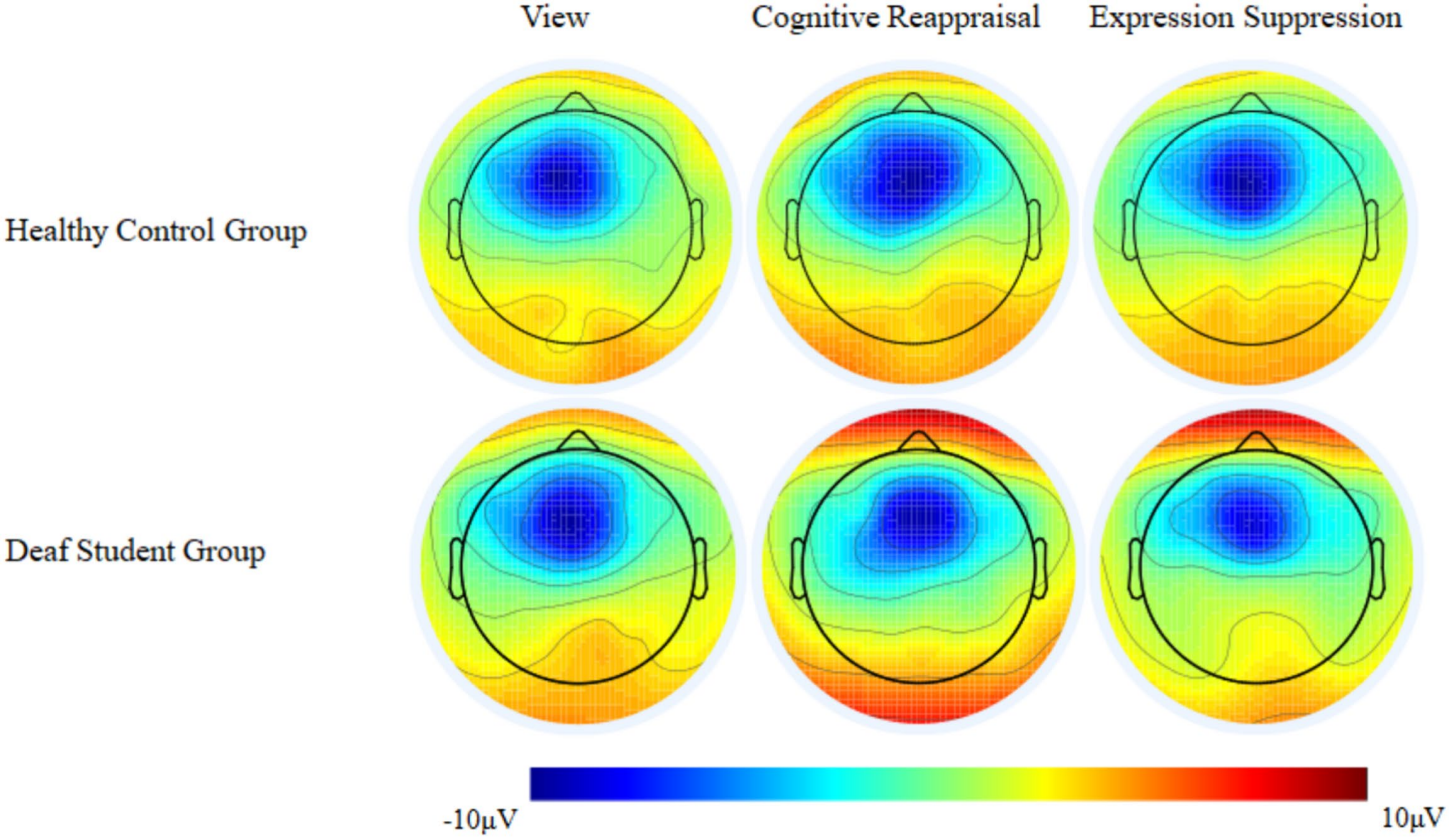
Scalp topography of LPP across three conditions at 420–620 ms.

For LPP amplitudes, we conducted a 2 (groups: deaf student group/healthy control group) × 3 (condition: passive view/reappraisal negative/suppression negative) repeated-measure ANOVA. The analysis showed a significant main effect of group (*F*_(1, 55)_ = 4.306, *p* = 0.043, *η_p_*^2^ = 0.073), condition (*F*_(2, 110)_ = 3.204, *p* = 0.048, *η_p_*^2^ = 0.106) (Figures 3, 4). Specifically, the control group (*M* = 3.461, SD = 0.335) elicited higher LPP than the deaf student group (*M* = 2.452, SD = 0.353), reappraisal negative (*M* = 2.818, SD = 0.283) and suppression negative (*M* = 2.649, SD = 0.242) were associated with a lower LPP amplitude than amplitudes associated with the passive view (*M* = 3.402, SD = 0.324) (Figure 2B).

Also, we observed a significant group × condition interaction (*F*_(2, 110)_ = 5.219, *p* = 0.008, *η_p_*^2^ = 0. 162), such that the healthy control group evidenced the LPP amplitudes were lower in the suppression negative than in the passive view and reappraisal negative condition, however, the deaf student group showed no differences.

To our surprise, we found that a correlation between depression scores and LPP amplitudes among the deaf college students. Pearson (*r*) correlation coefficients showed that decreases in depression scores were significantly correlated with the enlargement of LPP amplitudes for suppression condition (*r*_(27)_ = −0.422, *p* = 0.028).

## 4 Discussion

We used ERP to explore the characteristics of emotion regulation in deaf college students. The results indicated that the deaf student group exhibited higher emotional experience intensity rating scores and lower LPP amplitudes when reappraisal and suppression emotional stimuli were compared to the healthy control group. Furthermore, the LPP of suppression condition was associated with an increase in depression scores among deaf college students.

Emotional experience intensity scores indicated effective emotion regulation (Cai et al., 2016; Opoka et al., 2021). Compared to passive view, in behavioral results, both reappraisal negative and suppression negative resulted in significantly lower emotional experience intensity for all participants. Previous studies have also found that cognitive reappraisal and expression suppression were effective in reducing the participants’ negative emotions in healthy people (Cai et al., 2016).

But our results further found that deaf college students had higher emotional experience intensity than hearing college students under the two emotion regulation strategies. That is, deaf individuals showed weaker emotion regulation abilities when regulating negative emotions compared to the hearing person. These results supplied more evidence of children and adolescents with hearing loss have problems in managing and regulating their emotions compared to hearing ones (Adibsereshki et al., 2019; Ashori and Najafi, 2021; Dyck et al., 2004).

ERP results found that college students showed weaker LPP amplitude in response to negative emotional stimuli in the use of emotion regulation compared to regular college students. Many previous academic studies have confirmed the significant indicative role of EEG component LPP in emotional regulation through experimental data (Cai et al., 2016; Hajcak and Nieuwenhuis, 2006; Krompinger et al., 2008; Moser et al., 2006). Based on this conclusion, this study takes the EEG component LPP as one of the criteria to measure the effect of emotion regulation, which can reveal the shortcomings of deaf college students in emotion regulation compared with hearing college students. For example, fear faces activate higher LPP in children with hearing than in deaf children (Gu et al., 2019).

Studies have shown that LPP reflects the intensity of motivation but not the direction of motivation, but emotional valence is related to the direction of motivation, while negative emotion is related to avoidance of motivation (Bamford and Ward, 2008). Therefore, stronger the avoidance motivation for negative stimuli, the higher LPP amplitude is evoked (Leutgeb et al., 2009; Michalowski et al., 2009). Conversely, individuals with weaker avoidance motivation for negative stimuli had lower LPP amplitudes evoked by the stimuli (Li et al., 2022). Studies of depressed individuals have found reduced avoidance motivation for negative information (Li et al., 2022). This suggests that the deaf college students exhibited a reduced motivation to avoid negative stimuli. Consequently, they were unable to avoid negative stimuli and events in a timely manner, which in turn led to an increased experience of negative emotions in their lives. Studies on the ability of depressed patients to use specific emotional strategies have shown that, when guided, their ability to use cognitive reappraisal and expressive suppression to regulate their emotions does not differ significantly from that of hearing individuals (Ehring et al., 2010), and LPP amplitudes were significantly lower in the emotion regulation strategy condition than in the viewing condition (Zhang et al., 2016). However, our study found no significant difference in LPP amplitudes between the emotion regulation strategy condition and the viewing condition among deaf college students.

Previous studies have indicated that LPP amplitudes induced by emotional stimuli is negatively correlated with the level of depression (MacNamara et al., 2016; Weinberg et al., 2016; Klawohn et al., 2019; Nikolin et al., 2020). An indirect protective effect of suppression frequency on depression through suppression ability, that is, increased suppression frequency was associated with higher suppression ability, which in turn led to habitual use of suppression associated with fewer depressive symptoms (Chen et al., 2020). It indicated that among deaf college students, the easier it was to regulate negative emotions by using expression suppression strategies, the lower the level of depression. It may have something to do with specific cultural backgrounds (Butler et al., 2007). In East Asian cultures, expression suppression is associated with better social functioning. It is common and adaptively important, in life settings, for people to regulate negative and other maladaptive emotions by inhibiting emotion expressive behaviors, not only for keeping normal interrelation with other people, but also for the avoidance of violence, impulsive behavior, and other socially undesirable conducts (Davidson et al., 2000). Whose collectivistic cultural norms highlight the avoidance of hurting others, and the efforts to preserve and experience relational harmony (Soto et al., 2011; Yuan et al., 2015). We also found the deaf college students used expression suppression strategies more frequently to regulate negative emotions and habitually suppressed their emotions than the hearing college students. More fluent in the use of expression suppression strategies, the greater the suppression ability, therefore, the less likely to be depressed.

Actually, both results of behavioral ratings and LPP amplitudes in this study supported that the deaf college students have weaker motivation to avoid sad stimuli than hearing college students, which is consistent with previous studies (Grunewald et al., 2019). Behavioral results showed that compared with hearing college students, deaf college students experienced higher emotional intensity when using cognitive reappraisal and expression suppression strategies to regulate negative emotions. In other words, when deaf college students have difficult in controlling their behavior than hearing college students when experiencing negative emotions induced by negative events, and thus exhibit more irrational and impulsive behaviors (Zhang et al., 2012). ERP results showed that the LPP amplitude of deaf college students was significantly lower than that of hearing college students when they reevaluated and suppressed negative emotional stimuli, which was consistent with previous studies (Gu et al., 2019). The smaller the LPP amplitude, the weaker the avoidance motivation of negative stimulus (Leutgeb et al., 2009). In a word, when deaf college students encounter negative stimuli in daily life, they cannot avoid them in time, and are more likely to be involved in negative events, so they experience more negative emotions, which is also confirmed by behavioral results.

Even we have revealed the characteristics of ineffective emotion regulation in deaf college students by using ERP, there are still have the following limitations. Firstly, the sample size of this study is small, and the study object is only a single deaf college student, so the findings may not be generalized to other deaf groups. Future research could investigate the causes of developmental delays in deaf people by increasing the number of subject groups, as well as by considering variables that may be associated with difficulties in emotional regulation. Secondly, emotional intensity is one of the factors that influences the effectiveness of a regulatory strategy (Sheppes and Gross, 2013). Therefore, emotional intensity in the context of positive or negative images may affect the effectiveness of regulation strategies. Based on previous study (Hudak et al., 2022; Wang et al., 2015), only negative pictures were presented to induce participants’ negative emotions, and the influence of emotion regulation strategies on emotional experience and brain electrical activity was investigated. Therefore, in order to investigate whether the negative emotion regulation is impaired in deaf college students, so we only used negative pictures in our study. Certainly, in future study, we may use neutral pictures as control to further confirm the effect of negative faces. Finally, the ERP technique used in this study has high temporal resolution but poor spatial resolution and is unable to pinpoint the activation of brain regions. Therefore, future studies could incorporate additional techniques, such as fMRI, fNIRS, and transcranial magnetism, to conduct a more comprehensive investigation.

## 5 Conclusion

The results of this study suggested that deaf college students may have impaired emotion regulation compared to hearing college students, as evidenced by higher emotional experience intensity and evoked lower LPP amplitudes when using emotion regulation strategies. Moreover, the decrease in suppression condition LPP was associated an increase in depression scores among deaf college students. These results will be helpful in developing emotional regulation intervention programs for deaf college students.

## Data Availability

The original contributions presented in the study are included in the article/supplementary material, further inquiries can be directed to the corresponding author.
